# Paget's disease of nipple with dermal invasion: A case report

**DOI:** 10.1002/cnr2.1572

**Published:** 2021-10-27

**Authors:** Rubina Maharjan, Suraj Shrestha, Prafulla Shakya, Sanjeev Kharel, Aagon Krishna Shrestha, Moushami Singh

**Affiliations:** ^1^ Department of Oncology Nepal Cancer Hospital and Research Center Harisiddhi, Lalitpur Nepal; ^2^ Maharajgunj Medical Campus Institute of Medicine Maharajgunj, Kathmandu Nepal; ^3^ Department of Surgical Oncology (Breast Unit) Nepal Cancer Hospital and Research Center Harisiddhi, Lalitpur Nepal; ^4^ Department of Pathology Nepal Cancer Hospital and Research Center Harisiddhi, Lalitpur Nepal

**Keywords:** breast, DCIS, invasive Paget's disease, nipple

## Abstract

**Background:**

Invasive mammary Paget's disease (MPD) is an extremely rare eczematous eruption on the nipple and areola with an invasion of the dermis by Paget cells. This entity can often be misdiagnosed and overtreated for invasive carcinoma of the breast.

**Case:**

A 34‐year woman presented with a 2‐year history of right nipple eczema and right axillary lump for a month. Breast ultrasound revealed dilated intra‐nipple lactiferous duct and an enlarged right axillary lymph node. Histopathology from biopsy revealed MPD with ductal carcinoma in situ (DCIS) whereas final histopathology after right modified radical mastectomy revealed Invasive MPD with DCIS and axillary metastasis. She underwent adjuvant chemotherapy and is under hormonal therapy with complete remission for 18 months.

**Conclusion:**

Awareness of invasive MPD is important to avoid misdiagnosis and probable radical treatment. Close follow‐up is warranted due to limited knowledge regarding treatment and prognosis of invasive MPD.

## 1 INTRODUCTION

Paget's disease of the breast also known as mammary Paget's disease (MPD) accounts for about 1%–3% of primary breast cancer.^1^ An underlying invasive carcinoma or ductal carcinoma in situ (DCIS) is present in most of the MPD.^2^ MPD with nipple skin involvement and extending from lactiferous duct without basement membrane invasion is considered to be DCIS whereas invasion of tumor cells into the dermis by breaching basement membrane is considered invasive MPD.^2^ Invasive MPD is an extremely rare condition with only around 35 patients reported until date.^3^, ^4^, ^5^, ^6^ In addition, only six cases of invasive MPD with axillary node metastasis have been reported.^3^, ^4^

Here, we report a case of a 34‐year female who presented with nipple eczema and a right axillary lump for a month. Histopathology of the excised breast showed invasive MPD with lymph node metastasis.

## 2 CASE REPORT

A 34‐year female, P_3_L_2_D_1_, last childbirth 2 years back, presented with the complaint of right nipple eczema for 2 years and right axillary lump for 1 month. Initially, she noticed skin peeling off her right nipple associated with a pricking sensation. The lesion gradually increased involving the whole nipple associated with darkening of skin and irritation. There was no history of discharge, bleeding, or pruritus from the right nipple and it was unrelieved by any topical medications. She had no history of breast trauma. In addition, she noticed a lump in her right axilla for one month, gradually increasing in size and without any overlying skin changes. She had menarche at the age of 13 years with a regular cycle of 28–30 days. She breastfed all her children for 2 years. There was no history of use of any hormonal contraceptives and she underwent tubal ligation during her last childbirth. Moreover, there is no family history of breast, ovarian, and prostate cancer.

On examination, she had an eczematous lesion confined to the right nipple associated with darkening of skin without visible discharge. In the right axilla, a single, mobile, hard, non‐tender mass of about 1 cm was present. Left breast and left axillary examination were normal. Examination of other systems was unremarkable and all the laboratory investigations were within normal range.

Ultrasound of the right breast and axilla showed dilated intra‐nipple lactiferous duct showing sludge and an enlarged right axillary lymph node (30 mm × 12 mm) with loss of fatty hilum. The left breast had a normal scan with normal axilla. Due to inconclusive findings, a full‐thickness wedge biopsy from the right nipple and excision biopsy of a right axillary lymph node was done. A diagnosis of Paget's disease of the right breast with lymph node involvement was established.

**FIGURE 1:**
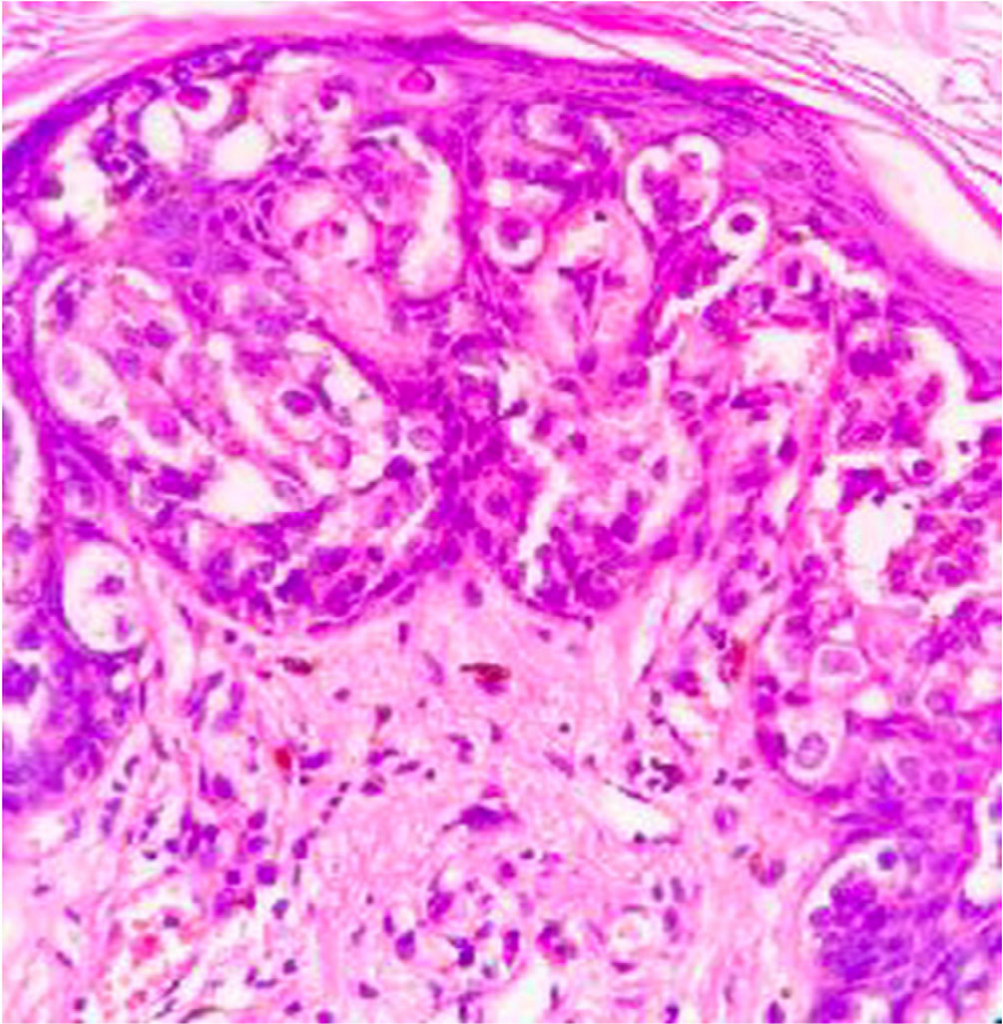
Microscopic image shows intraepithelial Paget's cells in clusters and singly having large nuclei with abundant pale cytoplasm beneath the epidermis

**FIGURE 2:**
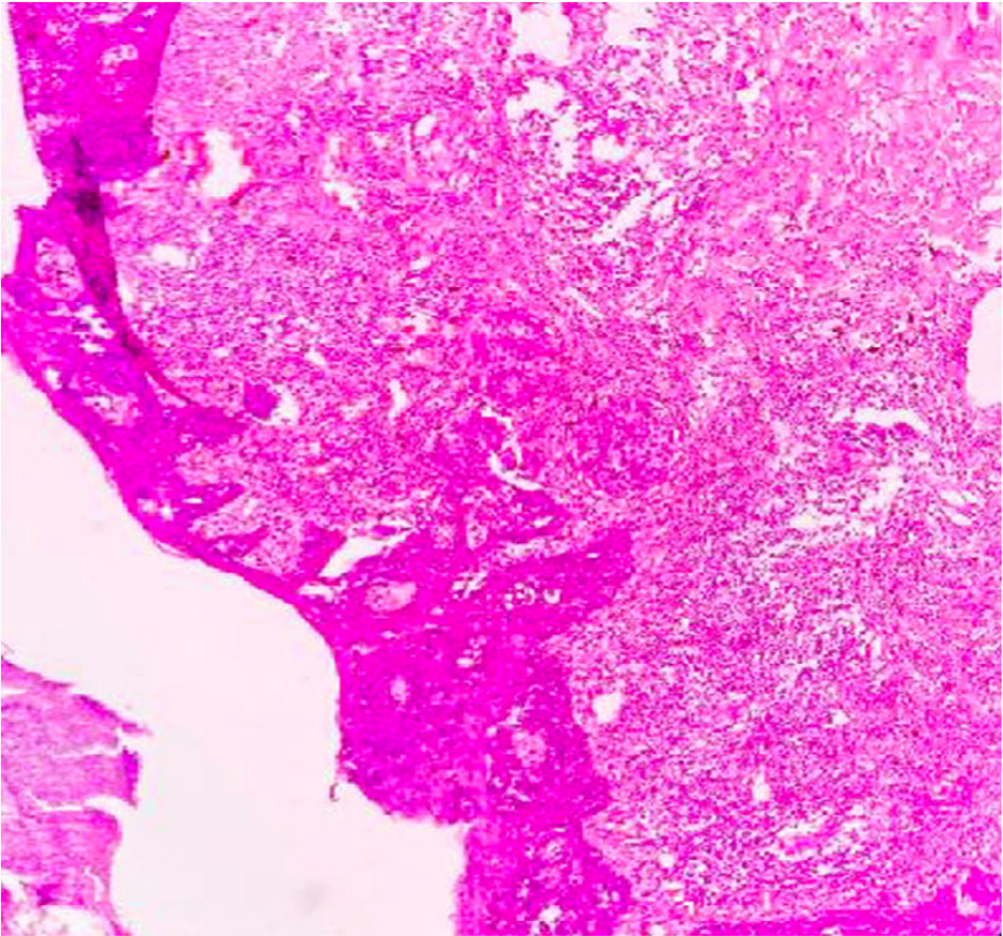
Histological section shows invasive Paget's disease with dermal invasion with tumor cells

**FIGURE 3:**
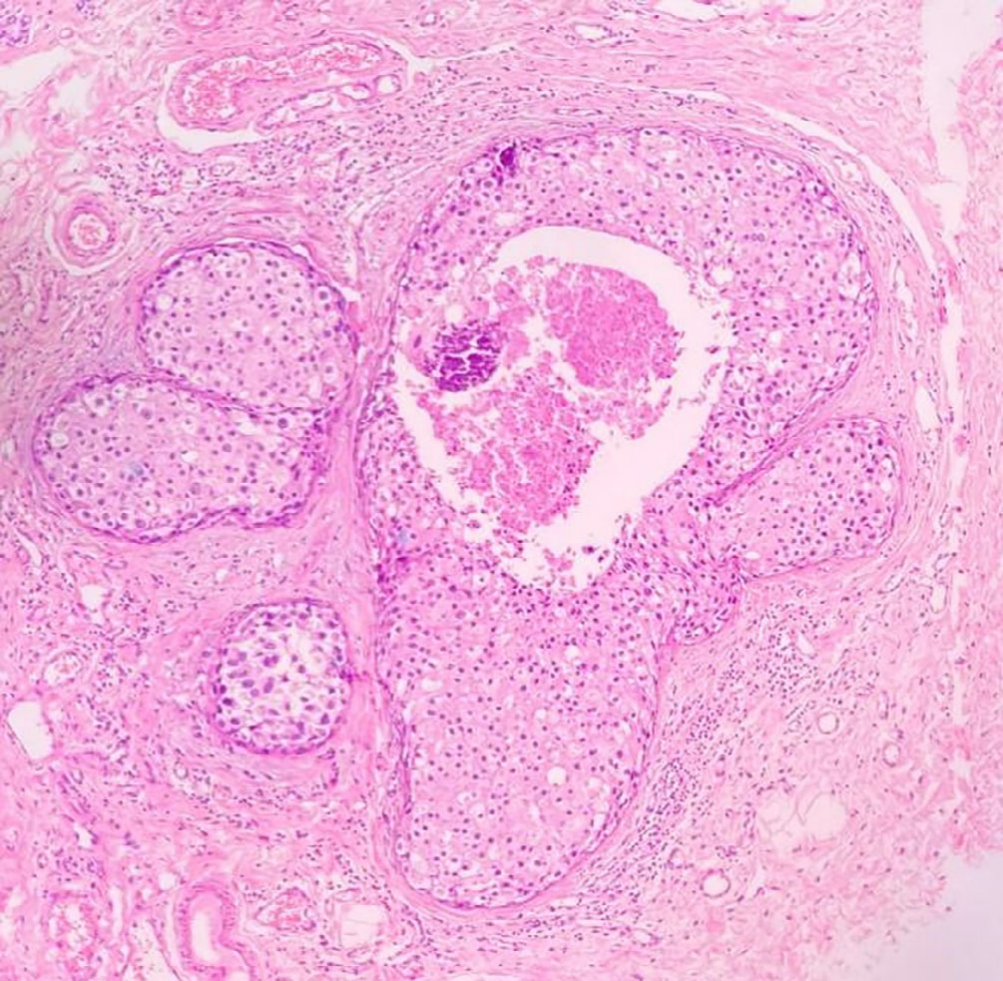
Histopathological section showing DCIS with central comedo necrosis

**FIGURE 4:**
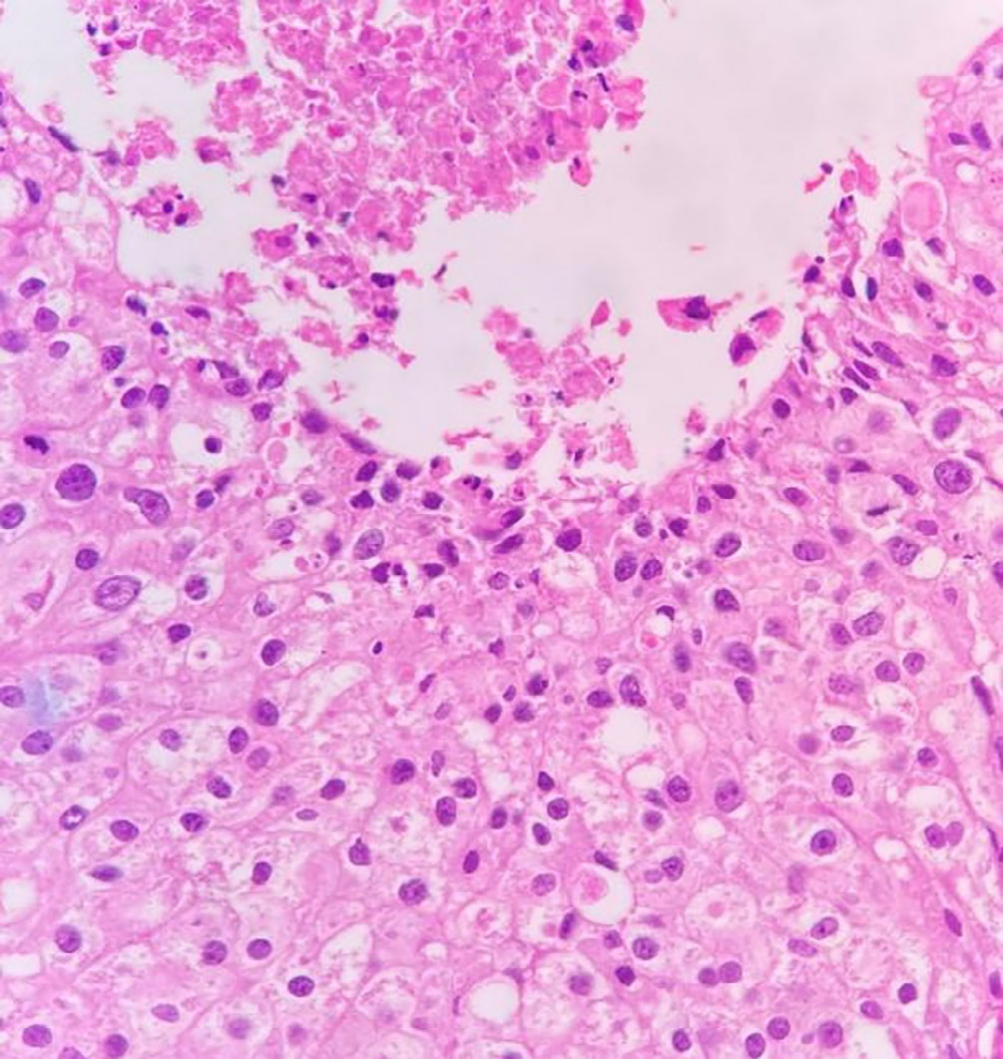
Histopathological section showing DCIS with microcalcification

The patient was 34 years old, so an MRI breast was planned. Due to financial reasons, she refused MRI breast. Following this, a contrast‐enhanced CT chest and abdomen was done which did not reveal any lesion in the left breast. No distant metastasis was found. Thus, the patient underwent a right modified radical mastectomy (MRM). However, the final histopathological reports from the right breast nipple showed features of Invasive Paget's disease of the nipple. The epidermis showed clusters and scattered cells with abundant pale cytoplasm, irregular large nuclei, prominent nucleoli in the epidermis (Figure 1). Clusters of the cells were seen infiltrating the dermis with a depth of invasion of 0.1 cm (Figure 2). Areas of DCIS were noted. No evident features of invasive ductal carcinoma were identified in several sections studied. Seven lymph nodes from the axillary lymph node of MRM specimen and five lymph nodes submitted separately as right axillary lymph node previously were noted. Hence, a total of 12 lymph nodes were identified and 1 lymph node was involved by the metastatic deposit. On immunohistochemistry performed on a metastatic axillary lymph node, tumor cells were positive for ER, negative for PR, positive for Her2Neu with a Ki67 proliferation index of 50%. Further histological analysis showed cribriform, micropapillary pattern with focal necrosis and microcalcifications, and Grade II (Intermediate) nuclear grade. (Figures 3 and 4) Multifocal DCIS was noted with the largest focus of DCIS measured 0.4 cm × 0.2 cm with ~0.1 cm away from the nipple. In addition, extensive grossing of the mastectomy specimen and histological section examination was done to rule out any invasive lesion in the breast.

The tumor board discussion was done and considering nodal positivity, she received 4 cycles of adriamycin plus cyclophosphamide followed by 4 cycles of paclitaxel‐based adjuvant chemotherapy with no adjuvant radiation therapy. The patient did not experience major side effects from chemotherapy except mild nausea and vomiting for a few days. As the patient could not afford HER2‐directed treatment, she is currently under tamoxifen 20 mg daily and on regular follow‐up, and under complete remission since 18 months of surgery.

## 3 DISCUSSION

Paget's disease of nipple with dermal invasion is an extremely rare condition as compared with extramammary Paget's disease with dermal invasion, which is a well‐documented entity. The prevalence of dermal invasion of Paget cells is around 4% to 7.8% of all cases of MPD.^3^, ^7^ Invasive MPD may not be noticed at the time of diagnosis, or may be misdiagnosed as an extension of invasive carcinoma to the skin, hence the notion of invasive MPD being rare has been questioned.^3^ Our patient was diagnosed with Paget's disease of the nipple with underlying DCIS before she underwent the right MRM.

Different theories have been put forward to explain MPD. Epidermotropic theory suggests the skin lesion of MPD is due to the migration of Paget's cells from underlying in situ or invasive carcinoma via a lactiferous duct into the nipple epidermis whereas in situ malignant transformation theory suggests the epidermal cells of the nipple undergo malignant transformation. Epidermotropic theory is most widely accepted due to the presence of underlying carcinoma in most cases of MPD. The absence of in situ and invasive carcinoma in breast parenchyma has been noted in a small subset of MPD cases suggesting an alternative etiology.^5^, ^8^ The largest study providing a clinicopathological profile of 16 patients with invasive MPD showed the mean age of diagnosis 47.6 years (35–76 years) with all female patients. Scaly eczematous skin change was the most common presenting symptom in the study with nipple discharge as another predominant symptom. The majority of the patients with skin changes had a palpable breast mass. Additionally, invasive ductal carcinoma was seen in the majority of patients. DCIS was found in a few patients and patients with no underlying breast pathology were very rare. PR status is found to be positive in a significantly higher number of cases with invasive MPD as compared to noninvasive MPD.^3^ Our patient was a 34‐year female who presented with an eczematous lesion and a palpable lymph node with an underlying DCIS. However, our patient was PR‐negative.

Due to the presence of underlying invasive breast malignancy in the majority of cases of MPD, a careful nodal examination is mandatory. For this very reason, further evaluation of any suspected case should be done. Mammography or biopsy can be done in the beginning.^9^ Invasive MPD is not usually associated with nodal involvement. However, our patient had a palpable right axillary node.

Moreover, the American Joint Committee on Cancer (AJCC) staging system classifies the disease stage of patients with invasive carcinoma and breast skin involvement as T4b. Skin involvement can be in the form of ulceration, edema, or satellite nodules. Invasive MPD should not be regarded as T4b as the regional or distant disease is not present in the case of dermal invasion by epidermal Paget cells. Hence, most of the reported cases are either T1mi (microinvasion) or T1a.^10^ Since the treatment and prognosis of these two entities are different, each case should be carefully evaluated and staged accordingly.^11^ Our patient had underlying DCIS and nodal positivity, however, was negative for any other organ metastasis.

Histopathologically, invasive MPD and skin involvement of breast cancer with pagetoid spread can have similar findings.^3^ Dermal invasion is an important histopathological criterion for the diagnosis of invasive MPD like extramammary Paget's disease.^7^ Pathologically, isolated or clustered Paget cells that have invaded the dermal‐epidermal junction is the characteristic feature of MPD with dermal invasion.^12^, ^13^ If the depth of invasion is <1 mm from the dermal‐epidermal junction or basement membrane, invasive MPD is again known as minimally invasive MPD.^14^ However, a distinction should always be made between secondary Paget's disease and Paget's disease with dermal invasion. Secondary Paget's disease occurs when skin is invaded by underlying invasive carcinoma due to infiltration of the epidermis by tumor cells. Such infiltration can be seen in the skin of the breast including other metastatic sites, and with carcinomas arising from organs other than the breast. In such cases, a large palpable mass is often found beneath the ulcerated area. A small superficial biopsy and inadequate or no clinical history is the only situation when a secondary Paget's disease can be confused with primary Paget's disease with dermal invasion.^15^ In general, postoperative tissue findings are often required for the diagnosis of intramammary Paget's disease rather than clinical symptoms. Similarly, the diagnosis was only made postoperatively in our case too.

Treatment of MPD with dermal invasion should be the same as the treatment for noninvasive Mammary Paget's Disease as there is no significant differences between patients with invasive and non‐invasive MPD with regard to underlying cancer status, immunohistochemical results for ER and HER2, immunohistochemical subtype classification, treatment, overall, and disease‐free survival.^3^ No distinguishing criteria have been yet known for the difference in the surgical management of MPD and MPD with dermal invasion. Local management of tumors is the most common treatment in invasive MPD. Hormonal therapy, if needed, is the only systemic treatment. In addition, there is no report of local or distant recurrences in the literature.^15^ In our case, we performed a right MRM due to the presence of lymph node involvement. Though the pathological staging showed Paget's disease with DCIS, the histopathological examination after surgery confirmed the diagnosis of Paget's disease of nipple with dermal invasion.

In general, skin invasion in breast cancer is a poor prognostic factor. However, invasive MPD is considered to have a favorable prognosis. However, the prognosis and clinical significance of invasive MPD are not well known due to a limited number of cases reported in the literature. As previous reports consistently suggested that the dermal invasion by Paget cells in invasive MPD did not influence the prognosis, and depends on the underlying carcinoma.^3^, ^7^, ^15^

## 4 CONCLUSION

Awareness of invasive MPD can help prevent clinicians from overlooking MPD invasive foci and reduce misdiagnosis of other types of invasive carcinomas can help prevent overdiagnosis and overtreatment. Moreover, close clinical follow‐up in this group of patients is imperative due to the limited information regarding its clinical and prognosis significance.

## CONFLICT OF INTEREST

The authors have stated explicitly that there are no conflicts of interest in connection with this article.

## AUTHOR CONTRIBUTIONS

**Rubina Maharjan**: Conceptualization‐Supporting, Resources‐Equal, Writing‐original draft‐Lead, Writing‐review & editing‐Equal. **Suraj Shrestha**: Conceptualization‐Equal, Investigation‐Equal, Writing‐review & editing‐Supporting. **Prafulla Shakya**: Conceptualization‐Lead, Formal analysis‐Lead, Supervision‐Lead, Writing‐review & editing‐Supporting. **Sanjeev Kharel**: Conceptualization‐Equal, Investigation‐Equal, Writing‐review & editing‐Supporting. **Aagon Shrestha**: Conceptualization‐Supporting, Supervision‐Supporting. **Moushami Singh**: Investigation‐Lead, Resources‐Equal, Writing‐review & editing‐Supporting.

## ETHICS STATEMENT

Not applicable.

## CONSENT FOR PUBLICATION

Written informed consent was obtained from the patient before the submission of the report. The signed Institutional Consent Form is on file.

## Data Availability

Data sharing is not applicable to this article as no new data were created or analyzed in this study.
